# Tidal changes in PaO_2_ and their relationship to cyclical lung recruitment/derecruitment in a porcine lung injury model

**DOI:** 10.1016/j.bja.2018.09.011

**Published:** 2018-11-03

**Authors:** D.C. Crockett, J.N. Cronin, N. Bommakanti, R. Chen, C.E.W. Hahn, G. Hedenstierna, A. Larsson, A.D. Farmery, F. Formenti

**Affiliations:** 1 Nuffield Division of Anaesthetics, University of Oxford, Oxford, UK; 2 Centre for Human and Applied Physiological Sciences, King's College, London, UK; 3 Vagelos College of Physicians and Surgeons, Columbia University, New York, NY, USA; 4 Hedenstierna Laboratory, Department of Medical Sciences, Uppsala University, Uppsala, Sweden; 5 Hedenstierna Laboratory, Department of Surgical Sciences, Uppsala University, Uppsala, Sweden; 6 Department of Biomechanics, University of Nebraska Omaha, Omaha, NE, USA

**Keywords:** diagnostic imaging, dynamic computed tomorgraphy, lung injury, pulmonary atelectasis, respiration, ventilation

## Abstract

**Background:**

Tidal recruitment/derecruitment (R/D) of collapsed regions in lung injury has been presumed to cause respiratory oscillations in the partial pressure of arterial oxygen (PaO_2_). These phenomena have not yet been studied simultaneously. We examined the relationship between R/D and PaO_2_ oscillations by contemporaneous measurement of lung-density changes and PaO_2_.

**Methods:**

Five anaesthetised pigs were studied after surfactant depletion via a saline-lavage model of R/D. The animals were ventilated with a mean fraction of inspired O_2_ (FiO_2_) of 0.7 and a tidal volume of 10 ml kg^−1^. Protocolised changes in pressure- and volume-controlled modes, inspiratory:expiratory ratio (I:E), and three types of breath-hold manoeuvres were undertaken. Lung collapse and PaO_2_ were recorded using dynamic computed tomography (dCT) and a rapid PaO_2_ sensor.

**Results:**

During tidal ventilation, the expiratory lung collapse increased when I:E <1 [mean (standard deviation) lung collapse=15.7 (8.7)%; *P*<0.05], but the amplitude of respiratory PaO_2_ oscillations [2.2 (0.8) kPa] did not change during the respiratory cycle. The expected relationship between respiratory PaO_2_ oscillation amplitude and R/D was therefore not clear. Lung collapse increased during breath-hold manoeuvres at end-expiration and end-inspiration (14% *vs* 0.9–2.1%; *P*<0.0001). The mean change in PaO_2_ from beginning to end of breath-hold manoeuvres was significantly different with each type of breath-hold manoeuvre (*P*<0.0001).

**Conclusions:**

This study in a porcine model of collapse-prone lungs did not demonstrate the expected association between PaO_2_ oscillation amplitude and the degree of recruitment/derecruitment. The results suggest that changes in pulmonary ventilation are not the sole determinant of changes in PaO_2_ during mechanical ventilation in lung injury.

**Editor's key points**

• Tidal recruitment/derecruitment is considered as a mechanism of ventilator-induced lung injury in acute respiratory distress syndrome.
• Respiratory oscillations in PaO_2_ have been hypothesised to be indicative of tidal recruitment/derecruitment.
• In a porcine model for lung injury, the authors investigated whether an association between tidal recruitment/decruitment and PaO_2_ oscillations existed.
• There was no change in amplitude of PaO_2_ oscillations during the respiratory cycle, and an association with tidal recruitment/decruitment was therefore not demonstrated.
• These findings challenge the hypothesis that respiratory oscillations in PaO_2_ are indicative of the lung collapse observed in lung injury.

Respiratory oscillations in the partial pressure of arterial oxygen (PaO_2_) have been hypothesised to indicate tidal recruitment/derecruitment (R/D) in acute respiratory distress syndrome (ARDS).^1^, ^2^ Notably, R/D is one of the proposed mechanisms of ventilator-induced lung injury in this condition.^3^, ^4^, ^5^ In addition, PaO_2_ oscillations *per se* could potentially cause or augment organ damage by exposing organs to cyclically varying O_2_ levels.^6^, ^7^, ^8^, ^9^ Despite the leading hypothesis that PaO_2_ oscillations are caused by tidal R/D, no study to date has demonstrated their relationship by simultaneous dynamic measurements of both PaO_2_ and R/D. Moreover, oscillations in PaO_2_ have been found in mechanically ventilated uninjured lungs with no tendency to collapse.^10^

Therefore, the aims of the present study were to explore whether R/D is the underlying reason for PaO_2_ oscillations by simultaneously measuring PaO_2_ and R/D dynamically in a porcine, collapse-prone ARDS model, and to examine whether larger increases in lung collapse during breath-hold manoeuvres are associated with a larger reduction in PaO_2_. For this purpose, we used newly developed fluorescence-quenching fibreoptic PaO_2_ probes with a response time of less than 100 ms that allow measurement of PaO_2_ oscillations in real time *in vitro* and *in vivo*^11^, ^12^, ^13^, ^14^ together with a single-slice dynamic CT (dCT) with a sampling interval of 250 ms affording measurements of R/D during mechanical ventilation.^15^

## Methods

### Ethical approval

This study of five domestic pigs (three males and two females; mean weight [standard deviation (sd)]=29.6 (1.7) kg) at the Hedenstierna laboratory, Uppsala University, Sweden was approved by the regional animal welfare ethics committee (Ref: C98/16) and adhered to Animal Research: Reporting of *In Vivo* Experiments guidelines.^16^ Measurements undertaken on the uninjured lungs of animals reported in this study have been published elsewhere.^10^

### Animal preparation

Table 1 shows the baseline characteristics of each animal. The animals were premedicated with i.m. xylazine 2 mg kg^−1^, ketamine 20 mg kg^−1^, and midazolam 0.5 mg kg^−1^, and underwent induction of anaesthesia with i.v. propofol titrated to effect (1–3 mg kg^−1^). The trachea was intubated and mechanical ventilation subsequently commenced. During preparation and before commencement of the study protocol, the animals were ventilated with volume-controlled ventilation (VCV) at 20–25 breaths per minute (bpm) [to maintain end-tidal CO_2_ (EtCO_2_) 4.5–6 kPa], with a tidal volume (*V*_*T*_) of 10 ml kg^−1^, positive end-expiratory pressure (PEEP) of 5 cm H_2_O, and an inspiratory:expiratory ratio (I:E) of 1:2. The ventilator tubing and tracheal tube were checked for leaks by analysis of the spirometry data. Anaesthesia was maintained with continuous i.v. ketamine 32 mg kg^−1^ h^−1^, fentanyl 4 μg kg^−1^ h^−1^, and midazolam 0.16 mg kg^−1^ h^−1^. General anaesthesia was confirmed by absence of spontaneous movements and by absence of reaction to painful stimulation between the front hooves. After confirmation of general anaesthesia, muscle relaxation was achieved with an initial bolus of rocuronium 0.2 mg kg^−1^ followed by 0.1 mg kg^−1^ boluses when spontaneous ventilatory efforts were detected from the airway gas and pressure traces. The adequacy of anaesthesia was determined during the periods of muscle relaxation by the absence of cardiovascular signs of sympathetic stimulation (increases in heart rate or arterial BP). Maintenance fluids were administered i.v. in the form of isotonic electrolyte solution (Ringerfundin; B. Braun Melsungen AG, Melsungen, Germany) at a rate of 10 ml kg^−1^ h^−1^ during the instrumentation phase and 7 ml kg^−1^ h^−1^ for the rest of the protocol. Once anaesthetised, bilateral surgical dissections of the neck were performed. The exposed right internal jugular vein was cannulated with a pulmonary artery catheter used for continuous pulmonary artery pressure monitoring, intermittent thermodilution cardiac output monitoring, and core temperature monitoring. The right and left internal carotid arteries were cannulated with 20G Leadercath Arterial cannulae (Vygon, Swindon, UK) for the introduction of fibreoptic PaO_2_ probes.

**Table 1:** Baseline characteristics and post-injury blood gas data for each animal. Pre-lavage blood gas values were within normal limits. Blood gas data presented were measured post-saline lavage. ♂, male; ♀, female. CO, cardiac output; FiO_2_, fraction of inspired O_2_; Hb, haemoglobin; PFR, PaO_2_:FiO_2_ ratio; sd, standard deviation

Variable	Animal number					Mean (sd)
	1	2	3	4	5	
Sex	♂	♀	♂	♀	♂	—
Weight (kg)	31.1	29.0	29.8	31.2	26.7	29.6 (1.7)
FiO_2_	0.5	0.8	0.7	0.7	0.9	0.7 (0.1)
PFR	288	285	232	105	276	237 (77)
pH	7.29	7.35	7.32	7.23	7.37	7.31 (0.05)
PaO_2_ (kPa)	19.2	38.1	21.7	9.8	33.0	24.4 (11.2)
PaCO_2_ (kPa)	8.2	7.3	7.8	8.9	6.8	7.8 (0.8)
Hb (g L^−1^)	91	80	81	86	76	82 (5)
CO (L min^−1^)	3.7	4.8	3.5	4.2	3.2	3.9 (0.6)

### Lung injury

A collapse-prone lung injury was induced with a technique modified from Lachmann and colleagues.^17^ Preoxygenation with a fraction of inspired O_2_ (FiO_2_) of 1.0 preceded the ventilator disconnection and lavage of the lungs by instillation of 0.9% saline solution (at 37°C) via the tracheal tube. After 30 s, the saline was drained out of the lungs and ventilation recommenced. This process was repeated until a PaO_2_:FiO_2_ ratio (PFR) of <300 mm Hg (40 kPa) was achieved.

### Data collection and processing

Cardiorespiratory variables, including peripheral O_2_ saturations (SpO_2_), ECG, invasive arterial BP (AS/3 Multi-Parameter Patient Monitor; Datex-Ohmeda, Madison, WI, USA), airway gas composition, flow, and pressure (Capnomac Ultima; Datex-Ohmeda), were continuously monitored and recorded as analogue signals throughout the protocol. PaO_2_ signals from the fibreoptic probes were continuously collected with OxyLitePro monitors (Oxford Optronix, Abingdon, UK), converted to digital form using PowerLab (ADInstruments, Dunedin, New Zealand) and displayed/recorded with LabChart version 8.1.5 (ADInstruments) with a sampling rate of 10 Hz. Physiological data were processed using R version 3.4.1 (R Core Team, Vienna, Austria).^18^

### Study protocol

The animals were positioned in dorsal recumbency on the CT scanner table. FiO_2_ [mean (sd)=0.7 (0.1)] was set depending on the lung injury achieved with saline lavages (see Table 1), and PaO_2_ was recorded continuously.

A first set of measurements considered tidal ventilation, when animals were ventilated in both pressure-controlled ventilation (PCV) and VCV modes at I:E ratios of 1:2, 2:1, 1:4, and 4:1 to explore the effects of different ventilatory modes on PaO_2_ and its dynamic changes. Upon each change of ventilator setting, the PaO_2_ trace was monitored until its mean value was stable, and then recorded for 120 s. The dCT images were recorded in the last 30 s of this period.

A second set of experiments considered breath-hold manoeuvres, when whole-lung CT scans were recorded during the first and last 5 s of an imposed 30 s breath hold at:

(i) End expiration: the expiratory and inspiratory valves closed at an initial airway pressure of 5 cm H_2_O airway pressure (Ve)
(ii) End inspiration: the valves closed after inspiration of *V*_*T*_ 10 ml kg^−1^ (*V*_*T*_10)
(iii) End large inspiration: the valves closed after inspiration of *V*_*T*_ 20 ml kg^−1^ (*V*_*T*_20).

Breath-hold manoeuvres were repeated multiple times in each animal in sequences designed to ensure all permutations of manoeuvre-order were achieved. The anaesthetised animals were euthanised with a bolus dose of potassium chloride (1–2 mmol kg^−1^) upon completion of the study protocol.

### CT image acquisition

A SOMATOM Definition Flash or SOMATOM Definition Edge (Siemens, Munich, Germany) were used to acquire all images as series of transverse sections with a reconstituted voxel size of 0.5 × 0.5 × 5 mm. Scans of a single juxta-diaphragmatic thoracic slice were acquired at 50 ms intervals with a 70 kV tube voltage, 246 mA current, and collimation of 64 × 0.6 mm in order to analyse dynamic changes during ventilation. A whole-lung scan was conducted at the start and immediately before the end of each breath-hold manoeuvre using a tube voltage of 80 kV, 364 mA current, and 64 × 60 mm collimation.

### CT image analysis

CT images were segmented using 3D Slicer version 4.6.2^19^ (http://www.slicer.org) with exclusion of the mediastinum, diaphragm, inferior vena cava, and hilar vessels. Exclusion of intrapulmonary vessels within regions of increased voxel density was not possible, and these, along with the conducting airways up to the level of the clavicles, were included in the analysis. Every fifth image was analysed producing a final temporal resolution of 250 ms. All images were then sub-segmented according to voxel density:^20^, ^21^, ^22^

(i) Collapse: –100 to +100 Hounsfield units (HU)
(ii) Poorly aerated lung: –500 to –101 HU
(iii) Normally aerated lung: –900 to –501 HU
(iv) Overdistended lung: –1000 to –901 HU.

The mass of each lung fraction (e.g. collapsed) was then calculated using the mean density and volume of each fraction assuming the lung is composed solely of air and water.^20^ The fractional mass of each region was then calculated as:

(1) (mass of fraction)*100%/(total mass of all fractions).

Tidal R/D was defined as the difference between the maximum and minimum measured mass of the collapsed lung during the course of a single breath.

### Statistical analysis

Statistical analyses were performed in GraphPad Prism (version 7.00 for Windows, GraphPad Software; La Jolla, CA, USA; https://www.graphpad.com). Before analysis, all data were tested for normality and homogeneity of variance. Parametric data were compared with paired, two-tailed, Student's *t*-test, and non-parametric with Wilcoxon matched-pairs signed-rank test. The level of significance was set at *P*<0.05 for all tests.

#### Tidal ventilation

CT measurements from all animals were compared using a one-way analysis of variance (anova) with multiple comparisons and Greenhouse–Geisser correction (parametric), or using a Wilcoxon matched-pairs signed-rank test (non-parametric). A two-way anova with Šidák correction for multiple comparisons was used for the analysis of CT measurements of inter- and intra-animal variability during tidal ventilation under different conditions.

#### Breath-hold manoeuvres

The effect of type of breath-hold manoeuvre on the change in lung collapse was compared using a Kruskal–Wallis test with Dunn's correction for multiple comparisons. A two-way anova was used to examine the effects of individual animal and type of breath-hold manoeuvre on the change in PaO_2_. Spearman correlations were used to assess the relationship between change in collapse and change in PaO_2_.

## Results

### Tidal R/D measured by dCT was detected when the expiratory time exceeded the inspiratory time during tidal ventilation

**Fig 1:**
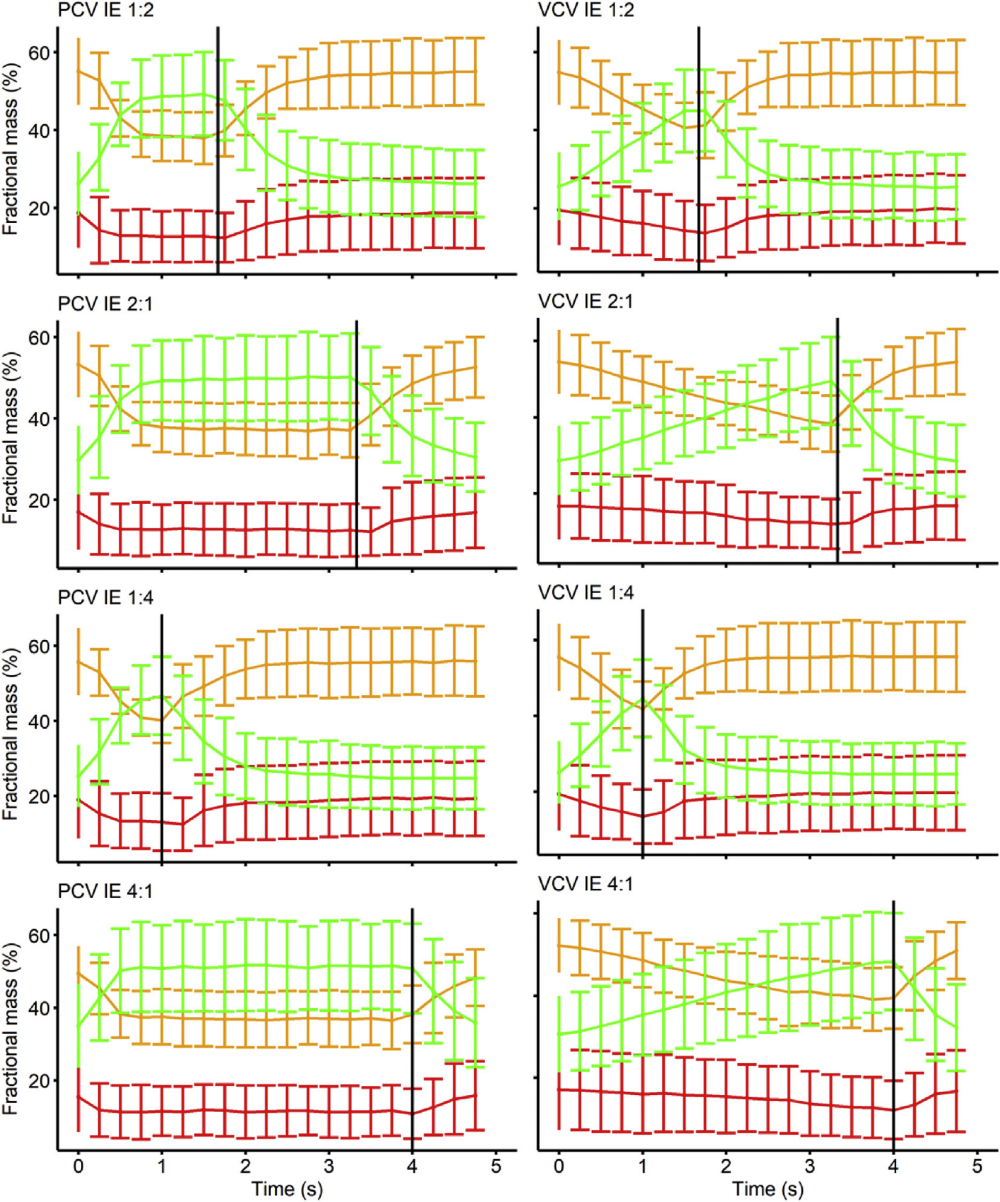
Changes in compartmental mass over the course of a single breath. Red, atelectasis; yellow, poorly aerated; and green, normally aerated. I:E, inspiratory:expiratory ratio; PCV, pressure-controlled ventilation; VCV, volume-controlled ventilation. Error bars represent standard deviation. Only in conditions where the expiratory time exceeded inspiratory time there was a significant difference between the mean maximum and minimum fractions of collapse, PCV 1:2 (11.6–19.5%), PCV 1:4 (11.5–19.9%), VCV 1:2 (12.6–20.4%), VCV 1:4 (12.9–20.6%). Overdistended mass represented <2% of total mass and remained unchanged throughout the breath in all conditions (not shown).

Figure 1 shows the changes in compartmental mass over the course of a single breath. The maximum fraction of collapse was significantly larger than the minimum fraction when I:E was below 1 [PCV 1:2 (11.6–19.5%), PCV 1:4 (11.5–19.9%), VCV 1:2 (12.6–20.4%), VCV 1:4 (12.9–20.6%); *P*<0.05], as quantified by single-slice dCT at a temporal resolution of 250 ms. This effect was consistent between PCV and VCV. There was no difference between the maximum and minimum fractions of collapse when I:E was higher than 1.

### Respiratory PaO_2_ oscillation amplitude was not clearly related to R/D

**Fig 2:**
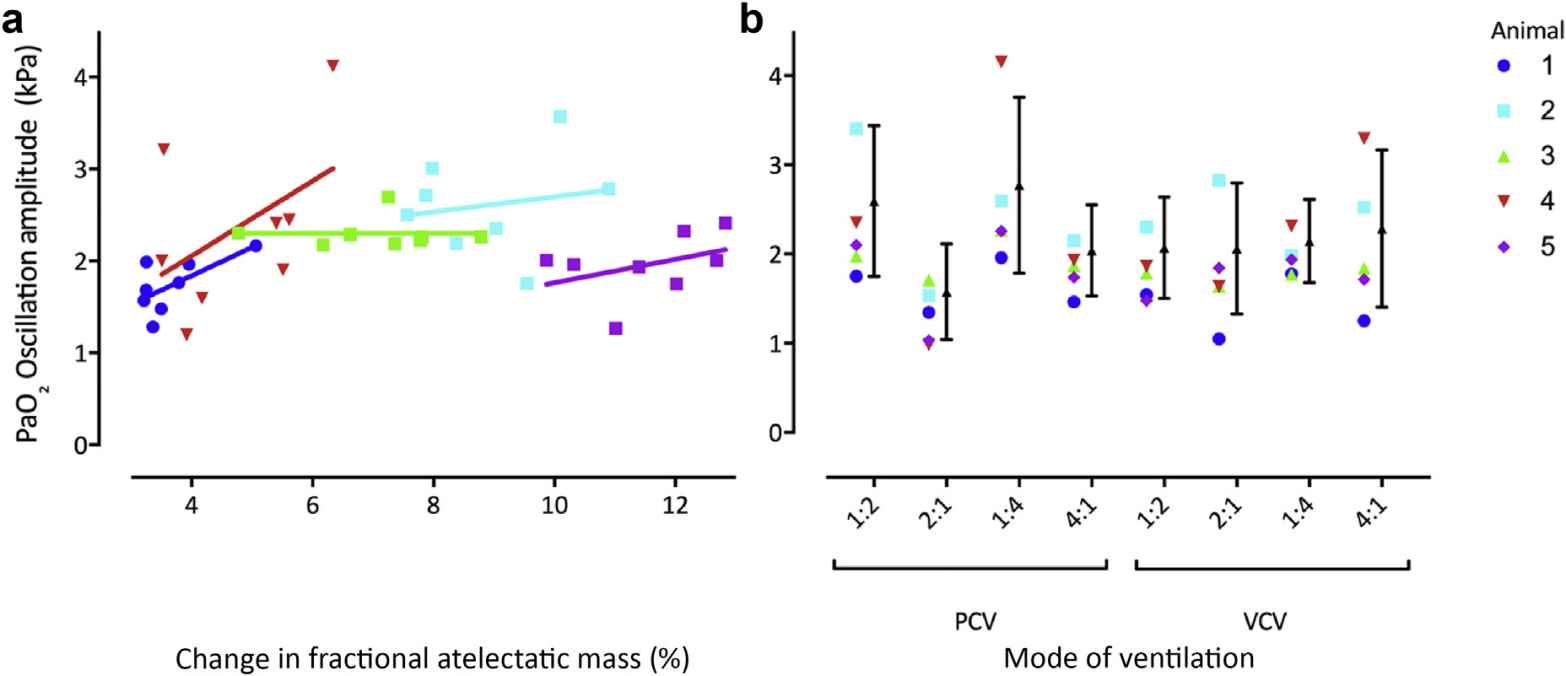
Mean respiratory PaO_2_ oscillation amplitude during tidal ventilation under different ventilatory conditions. (a) Correlation between the mean respiratory PaO_2_ oscillation amplitude (kPa) recorded during CT scanning and the relevant associated CT-measured change in fractional collapse during that ventilatory condition. The linear regression analysis results gave: Pig 1: *r*^2^=0.44, gradient=2.33; Pig 2: *r*^2^=0.31, gradient=0.62; Pig 3: *r*^2^=0.00, gradient=0.00; Pig 4: *r*^2^=0.23, gradient=3.06; Pig 5: *r*^2^=0.15, gradient=0.96. (b) Mean amplitude (kPa) with error bars representing standard deviation (black dots and lines). Amplitudes are calculated from tidal ventilation both before and during CT for each ventilator condition. Each animal is represented by a different coloured symbol. I:E, inspiratory:expiratory ratio; PCV, pressure-controlled ventilation; VCV, volume-controlled ventilation; x-axis ratios in (b) represent different I:E ratios.

A total of *n*=148 different 30 s sections of respiratory PaO_2_ oscillation data were analysed, all of which were with *V*_*T*_=10 ml kg^−1^ and ventilatory frequency=12 bpm. Figure 2a illustrates the relationship between the change in lung collapse and the respiratory PaO_2_ oscillation amplitude. The mean PaO_2_ and respiratory PaO_2_ oscillation amplitude for each ventilator condition are shown in Table 2 and Figure 2b. There was not a strong correlation between mean airway pressure and mean PaO_2_ during tidal ventilation, with a 0.55 kPa reduction in PaO_2_ for 1 cm H_2_O increase in airway pressure (*r*=−0.46). A one-way repeated measures anova with multiple comparisons supported a significant effect of ventilatory mode on mean PaO_2_ during tidal ventilation (*F*_(3,57)_=7.6; *P*=0.0002).

**Table 2:** Mean PaO_2_, amplitude of respiratory PaO_2_ oscillations, and mean airway pressure during different ventilatory conditions. Values shown are mean (standard deviation)

I:E ratio	Pressure-controlled ventilation			Volume-controlled ventilation		
	Mean PaO_2_ (kPa)	Oscillation amplitude (kPa)	Mean airway pressure (cm H_2_O)	Mean PaO_2_ (kPa)	Oscillation amplitude (kPa)	Mean airway pressure (cm H_2_O)
1:2	25.2 (4.7)	2.6 (0.8)	11 (8)	26.4 (4.6)	2.1 (0.6)	8 (5)
2:1	19.7 (6.6)	1.6 (0.5)	16 (8)	25.9 (5.5)	2.1 (0.7)	11 (6)
1:4	27.1 (2.5)	2.8 (1.0)	8 (7)	26.3 (3.6)	2.1 (0.5)	7 (5)
4:1	22.7 (3.9)	2.0 (0.5)	18 (6)	26.4 (3.2)	2.3 (0.9)	13 (5)

Respiratory PaO_2_ oscillation amplitude was significantly lower during PCV 2:1 when compared to PCV 1:2, PCV 1:4, VCV 1:2 and significantly higher during PCV 1:4 compared to PCV 4:1, PCV 2:1 and VCV 1:4.

### Whole-lung collapse did not decrease during a 30 s breath-hold manoeuvre with large tidal volume

The analysis of *n*=74 breath-hold manoeuvres undertaken with simultaneous CT showed a significant increase in the fraction of collapse from the start to the end of an imposed breath-hold manoeuvre, as shown in Table 3. The mean (sd) airway pressure decreased concurrently during breath-hold manoeuvres at end expiration by 4 (2) cm H_2_O, *V*_*T*_10 by 11 (6) cm H_2_O, and *V*_*T*_20 by 11 (6) cm H_2_O. The fractional increase in collapse during the end-expiratory breath-hold manoeuvres was significantly larger compared with the other two conditions (14% *vs* 0.9–2.1%; *P*<0.0001). There was no difference between the change in collapse with *V*_*T*_10 and *V*_*T*_20 end-inspiratory breath-hold manoeuvres. The change in PaO_2_ could not be predicted from the change in collapse with simple linear regression (*r*^2^=0.23, 0.15, 0.14 for Ve, *V*_*T*_10, and *V*_*T*_20 breath-hold manoeuvres, respectively).

**Table 3:** Mean fractional mass of collapsed lung measured by CT at the start and end of breath-hold manoeuvres. Ve, end expiratory; *V*_*T*_10, end-inspiratory (10 ml kg^−1^); *V*_*T*_20, end-inspiratory (20 ml kg^−1^). ^∗^Ve: *t*(24)=12; *P*<0.0001. ^†^*V*_*T*_10: *t*(24)=3.2; *P*<0.005. ^‡^*V*_*T*_20: 95% confidence interval: 0.6–1.5%

Animal number	Fractional collapse (%)					
	Ve		*V*_*T*_10		*V*_*T*_20	
	Start	End	Start	End	Start	End
1	18.4 (2.1)	23.8 (2.0)	16.8 (1.4)	17.5 (2.1)	15.3 (1.7)	16.0 (1.3)
2	37.1 (2.9)	55.3 (3.6)	22.5 (7.7)	28.3 (9.4)	13.0 (2.3)	15.8 (1.8)
3	25.4 (1.1)	37.8 (1.4)	19.9 (0.7)	22.5 (0.5)	16.1 (0.6)	17.0 (1.4)
4	19.8 (1.2)	36.8 (3.0)	8.7 (1.6)	10.2 (2.5)	7.0 (0.6)	9.6 (3.2)
5	32.8 (0.9)	53.1 (0.7)	25.6 (3.4)	27.1 (0.7)	19.1 (1.1)	20.0 (1.4)
Combined	26.0 (7.1)	40.1 (12.0)	18.6 (6.5)	20.7 (7.1)	14.8 (4.1)	16.1 (3.7)
Difference	14.1^∗^ (6.1)		2.1^†^ (3.0)		0.9 (0.6–1.5)^‡^	

### Significant variation in the PaO_2_ change for different breath-hold manoeuvres within and between individual animals

**Fig 3:**
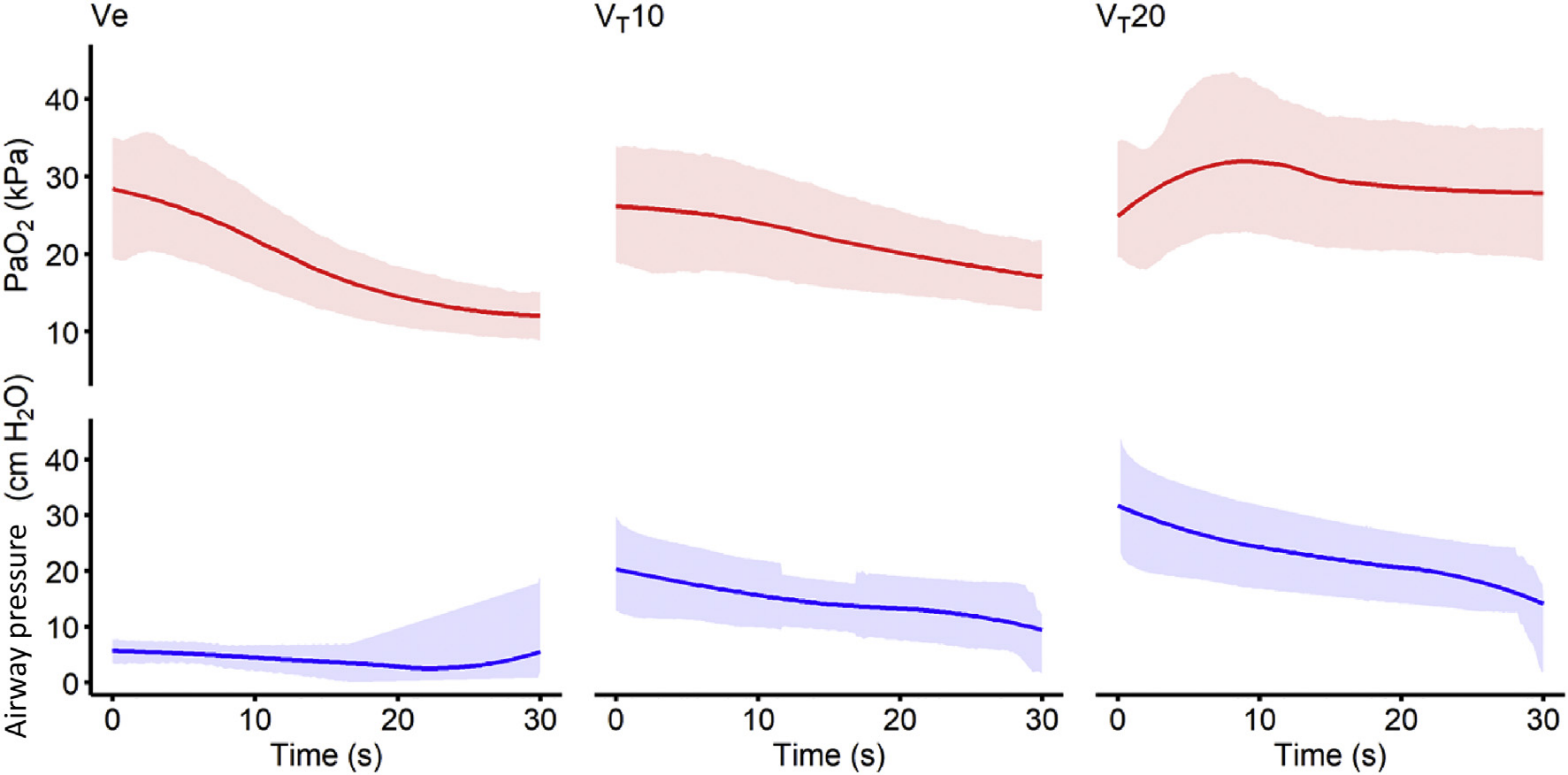
PaO_2_ and airway pressure traces during breath-hold manoeuvres. The left column shows end-expiratory breath-hold manoeuvres (Ve), the middle column 10 ml kg^−1^ end-inspiratory breath-hold manoeuvres (*V*_*T*_10), and the right column 20 ml kg^−1^ end-inspiratory breath-hold manoeuvres (*V*_*T*_20). Red represents PaO_2_ and blue represents mean airway pressure. Solid lines represent mean of *n*=5 animal manoeuvres associated with CT imaging. The shaded area represents standard deviation. PaO_2_ traces have been corrected for the effect of O_2_ uptake ($\dot{\text{V}}$O_2_) over time.

Figure 3 shows the mean continuous PaO_2_ recordings from the animals (*n*=5) during each breath-hold manoeuvre studied before and during CT scanning (*n*=146). For each individual manoeuvre, the change in PaO_2_ was calculated from the start of the breath-hold manoeuvre (measured from the start of the imposed airway pressure change) to the subsequent nadir in the trace. The mean (sd) change in PaO_2_ was –16.5 (6.3) kPa during end-expiratory breath-hold manoeuvres, and –10.5 (5.0) kPa and –4.8 (3.4) kPa for *V*_*T*_10 and *V*_*T*_20 end-inspiratory breath-hold manoeuvres, respectively. A repeated measures anova with Greenhouse–Geisser correction determined that these PaO_2_ changes were significantly different between each condition (*F*_(2,38)_=110; *P*<0.0001). Supplementary Table S1 shows *post hoc* multiple comparisons, and Supplementary Figure S1 shows all recorded traces for each animal and breath-hold manoeuvre. These demonstrate high variability between each animal and manoeuvre in the majority of cases.

## Discussion

This study investigated the relationship between dynamic changes in PaO_2_ and collapse in anaesthetised, mechanically ventilated pigs with saline-lavage lung injury. We demonstrated dynamic respiratory PaO_2_ oscillations during tidal ventilation, which increased when CT markers of tidal R/D increased. However, the magnitude of this association was not as large as expected. We found a significant tidal R/D only when I:E <1. Additionally, our study showed an increase in lung collapse even during a 30 s large inspiratory breath-hold manoeuvre and that the increase in collapse was associated with a significant reduction in PaO_2_ during these manoeuvres, including when the effect of continuous O_2_ uptake ($\dot{\text{V}}{\text{O}}_{2}$) was considered.

The analysis of the respiratory PaO_2_ oscillation amplitude showed that there were differences in the amplitudes for some conditions; however, these did not match the conditions, in which R/D was detected by dCT. The minimum and maximum amounts of mean fractional collapse (mean (SD)) were 14 (7) % and 17 (9) % measured in PCV 2:1 and PCV 1:4 respectively. Given the small differences and large variability in these values, it is likely that, whilst technically measurable, they do not represent a meaningful physiological or clinical difference. These results suggest a lack of a strong association between R/D and respiratory PaO_2_ oscillations. This finding does not support the hypothesised strong causal relationship between them,^1^, ^2^, ^23^, ^24^ and suggests the presence of other contributing determinants of variable shunt fraction within each breath. This proposition is supported by results from studies in the uninjured porcine lung, where the presence of respiratory PaO_2_ oscillations was demonstrated in the absence of R/D.^10^ The measured mass of collapse [mean (SD) = 16 (9)%] was lower than that reported in other studies using dCT^25^, ^26^, ^27^ in lung injury, although some of these studies considered different HU ranges. The measured collapse in our study, however, was 88 (59)% higher than that measured in studies examining a similar protocol in the uninjured lung using the same species and model,^10^ and consistent with findings from intra-vital microscopy.^28^ This result suggests that other determinants of respiratory PaO_2_ oscillations should be considered. Whilst R/D is likely to cause an increase in the amplitude of respiratory PaO_2_ oscillations, in the context of a complex physiological system, the effect may be obscured by other competing variables, such as the redistribution of pulmonary blood flow to regions with different ventilation:perfusion ratios.^29^ This hypothesis is supported by the finding in our study that mean PaO_2_ did not increase with an increase in mean airway pressure, as reported previously.^30^, ^31^ Additionally, CT-measured voxel density used as a surrogate marker of collapse assumes that the higherdensity voxels are true collapse and not another high radiodense material, such as fluid (alveolar flooding) or blood, and so may overstate the degree of true collapse.

The observed amplitude of oscillations was smaller than had been demonstrated elsewhere.^1^, ^2^ This may be partially explained by the smaller *V*_*T*_ (10 *vs* ∼30 ml kg^−1^) and lower peak *P*_AW_ (<35 *vs* >45 cm H_2_O) during tidal ventilation in our study. Whilst the *V*_*T*_ is larger than the recommended human clinical *V*_*T*_,^32^ it is smaller than those used in studies showing respiratory PaO_2_ oscillations with amplitudes of >13 kPa, and equivalent to *V*_*T*_ measured in spontaneously ventilating pigs.^33^ In addition, peak pressure was <30 cm H_2_O throughout the experiments, in contrast to previous studies where peak pressure exceeded 45 cm H_2_O.^1^

The amount of lung collapse, unexpectedly, did not decrease during imposed inspiratory (*V*_*T*_=10 and 20 ml kg^−1^) breath-hold manoeuvres. In the context of evidence demonstrating that the majority of recruitment occurs within the first few seconds of application of inflation pressure,^15^, ^33^, ^34^ this result may represent CT measurement of ‘starting’ collapse being taken at a time point already on the plateau of the recruitment curve. In fact, the starting collapse mass recorded in Ve was 31% greater than that recorded in *V*_*T*_20 (26.0–14.8%). However, it is important to recognise that the airway pressure is not maintained during a prolonged pause, as both the expiratory and inspiratory ventilator valves close at the start of manoeuvre, and the pressure in the lungs decreases as a result of the ‘pendelluft’ phenomena^35^ and oxygen consumption ($\dot{\text{V}}{\text{O}}_{2}$), although we attempted to correct for the effect of $\dot{\text{V}}{\text{O}}_{2}$ in our analysis by subtracting the calculated $\dot{\text{V}}{\text{O}}_{2}$ at each 100 ms time point. The reduction in airway pressure will increase both the amount of poorly aerated regions and lung collapse, which in turn will reduce PaO_2_ by increasing shunt and V/Q mismatch. Indeed, we found a decrease in airway pressure during all breath-hold manoeuvres.

The main limitations of our study are that the porcine model does not comprise all the features observed in human ARDS, and that the PFRs attained were consistent with only mild to moderate lung injury. However, the lavage model is very prone to collapse and is easily recruitable. Thus, this model of lung injury would exaggerate the R/D phenomena and possible R/D dependent PaO_2_ oscillations.

In conclusion, to the best of our knowledge, this is the first study to measure contemporaneously dynamic R/D and PaO_2_ in a collapse-prone ARDS model. We found a very limited association between R/D and respiratory PaO_2_ oscillations, certainly much smaller than expected from the published literature. These results challenge the accepted hypothesis that R/D is the main determinant of respiratory PaO_2_ oscillations in ARDS, where reduction of PaO_2_ oscillation amplitude is mostly expected from reduction of R/D. Our study warrants further investigation into the dynamic, often overlooked role of pulmonary perfusion within the complex context of pulmonary responses to mechanical ventilation.^10^, ^36^

## Authors' contributions

Study design: FF, GH, CH, AF.

Study conduct: FF, NB.

Data analysis: DC.

Data interpretation: DC, FF, JC, AF, AL, GH.

Writing of paper: DC, FF.

Critical revision: all authors.

Financial support: FF, AF, CH, AL.
