# Supplementary material for: Tidal changes in PaO2 and their relationship to cyclical lung recruitment/derecruitment in a porcine lung injury model
Source: Br J Anaesth. 2018 Nov 3;122(2):277–85. doi: 10.1016/j.bja.2018.09.011 (PMC6354046; doi:10.1016/j.bja.2018.09.011)
Supplement: Multimedia component 1 [file mmc1.docx]

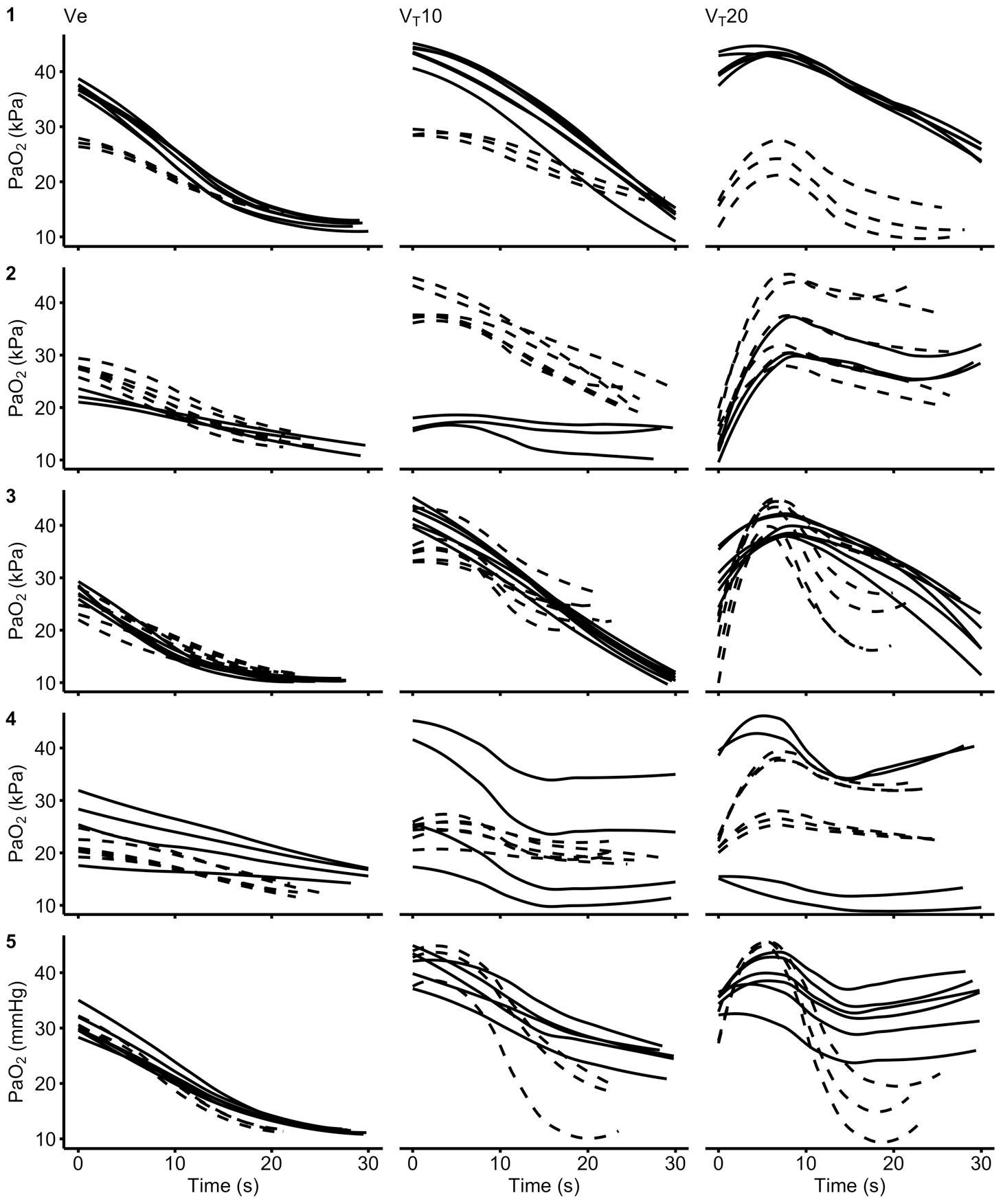


**Supplementary Figure 1 PaO_2_ traces during breath-hold manoeuvres for each animal.** The left column shows end-expiratory breath-hold manoeuvres (Ve), the middle column 10 ml kg^-1^ end-inspiratory breath hold-manoeuvres (V_T_10) and the right column 20 ml kg^-1^ end-inspiratory breath hold manoeuvres (V_T_20). Dashed lines represent measurements undertaken in the laboratory before transfer to the CT scanner. Solid lines are manoeuvres associated with CT imaging. Each animal is represented by the number to the left of each set of plots. All traces have been corrected for the effect of O_2_ uptake (V̇O_2_).
