# Supplementary material for: Tidal changes in PaO2 and their relationship to cyclical lung recruitment/derecruitment in a porcine lung injury model
Source: Br J Anaesth. 2018 Nov 3;122(2):277–85. doi: 10.1016/j.bja.2018.09.011 (PMC6354046; doi:10.1016/j.bja.2018.09.011)
Supplement: Multimedia component 2 [file mmc2.docx]

**Supplementary Table 1: Change in PaO_2_ during breath-hold manoeuvres in different animals.**

| Animal Comparison | Breath-hold Manoeuvre Type | | |
| --- | --- | --- | --- |
|  | Ve | V_T_10 | V_T_20 |
| 1 vs 2 | **** | **** | **** |
| 1 vs 3 | * | * | ns |
| 1 vs 4 | *** | **** | **** |
| 1 vs 5 | ns | **** |  |
| 2 vs 3 | ** | **** | **** |
| 2 vs 4 | ns | ns | ** |
| 2 vs 5 | **** | * | *** |
| 3 vs 4 | ns | *** | ** |
| 3 vs 5 | ns | * | ns |
| 4 vs 5 | ** | ns | ns |
| Ve = end-expiratory, V_T_10 = end-inspiratory (10 ml kg^-1^), V_T_20 = end-inspiratory (20 ml kg^-1^). Significant differences in the change in PaO_2_ are denoted against the relative comparison; * = p < 0.05, ** = p < 0.01, *** = p < 0.005, **** = p < 0.0001, ns = not significant. | | | |
